# Outbreak of colistin and carbapenem-resistant *Klebsiella pneumoniae* ST16 co-producing NDM-1 and OXA-48 isolates in an Iranian hospital

**DOI:** 10.1186/s12866-024-03207-6

**Published:** 2024-02-17

**Authors:** Rahimeh Sanikhani, Mojtaba Akbari, Majid Hosseinzadeh, Mansour Siavash, Farzad Badmasti, Hamid Solgi

**Affiliations:** 1 Department of Bacteriology, Pasteur Institute of Iran, Tehran, Iran; 2 Isfahan Endocrine and Metabolism Research Center, Isfahan University of Medical Sciences, Isfahan, Iran; 3 Department of Genetics and Molecular Biology, School of Medicine Isfahan University of Medical Sciences, Isfahan, Iran; 4 Department of Laboratory Medicine, Amin Hospital, Isfahan University of Medical Sciences, Isfahan, Iran

**Keywords:** Ventilator-associated pneumonia, Carbapenem-resistant *Klebsiella pneumoniae*, Colistin resistance, Carbapenemase genes, Sequence type 16

## Abstract

**Background:**

Colistin and carbapenem-resistant *Klebsiella pneumoniae* (Col-CRKP) represent a significant and constantly growing threat to global public health. We report here an outbreak of Col-CRKP infections during the fifth wave of COVID-19 pandemic.

**Methods:**

The outbreak occurred in an intensive care unit with 22 beds at a teaching university hospital, Isfahan, Iran. We collected eight Col-CRKP strains from seven patients and characterized these strains for their antimicrobial susceptibility, determination of hypermucoviscous phenotype, capsular serotyping, molecular detection of virulence and resistance genes. Clonal relatedness of the isolates was performed using MLST.

**Results:**

The COVID-19 patients were aged 24–75 years with at least 50% pulmonary involvement and were admitted to the intensive care unit. They all had superinfection caused by Col-CRKP, and poor responses to antibiotic treatment and died. With the exception of one isolate that belonged to the ST11, all seven representative Col-CRKP strains belonged to the ST16. Of these eight isolates, one ST16 isolate carried the *iucA* and *ybtS* genes was identified as serotype K20 hypervirulent Col-CRKP. The *bla*_SHV_ and *bla*_NDM-1_ genes were the most prevalent resistance genes, followed by *bla*_OXA-48_ and *bla*_CTX-M-15_ and *bla*_TEM_ genes. Mobilized colistin-resistance genes were not detected in the isolates.

**Conclusions:**

The continual emergence of ST16 Col-CRKP strains is a major threat to public health worldwide due to multidrug-resistant and highly transmissible characteristics. It seems that the potential dissemination of these clones highlights the importance of appropriate monitoring and strict infection control measures to prevent the spread of resistant bacteria in hospitals.

## Introduction

*Klebsiella pneumoniae* is a major Gram-negative bacterial pathogen that can cause invasive hospital-acquired infections among patients, especially those admitted to the intensive care unit (ICU) [1]. Carbapenem-resistant *K. pneumoniae* (CRKP) represents a major healthcare problem globally being associated with increased infectious morbidity and mortality due to limited treatment options [2]. Usually, resistance to carbapenems is mediated by carbapenemase production or by overexpression of AmpC cephalosporinases in combination with porin mutations. So far, the most common carbapenemases, in terms of carbapenem hydrolysis and geographical spread, are KPC, the MBLs NDM, VIM and IMP, and OXA-48 [3]. Carbapenem resistance has been described in many distinct *K. pneumoniae* genotypes determined with the help of multi-locus sequence typing (MLST). Carbapenemase-producing *K. pneumoniae* (CPKP) is often extensively drug-resistant (XDR) and poses serious problems in terms of clinical treatment and infection control. In recent years in Iran, OXA-48 and NDM-1 producing *K. pneumoniae* has been recognized, which belong to different clones (especially clones, ST11, ST893 and ST147) [4–6].

Colistin represents a few antimicrobials remaining active against infections caused by CPKP isolates. Colistin resistance in *K. pneumoniae* is mediated by several mechanisms. The most common strategies for resistance to colistin are modifications of the bacterial outer membrane through alteration of the lipopolysaccharid. The other mechanisms include overexpression of efflux-pump systems and point-mutations in *pmrB*, and *MgrB* genes. Another mechanism is overproduction of capsule polysaccharide. In addition, horizontal transfer of plasmid-mediated mobile colistin resistance gene, *mcr*, play a significant role in the dissemination of colistin resistance [7, 8]. The use of colistin as an option to treat infections caused by carbapenem-resistant Gram-negative bacteria has led to increased resistance to this antibiotic in recent years, which is now challenging the effectiveness of this therapy [9]. Since there are no novel β-lactam agents (i.e., meropenem-vaborbactam, imipenem-cilastatin-relebactam and cefiderocol) and tigecycline in the list of Iranian pharmacopoeias, colistin is almost the last resort of CPKP treatment in Iran. This may be due to the lack of extensive epidemiological studies on the prevalence of carbapenemase genes in our country. Although tigecycline and ceftazidime-avibactam can be obtained freely in the market recently, it cannot be obtained for many patients due to high costs and lack of insurance coverage. Therefore, increasing resistance to colistin in CPKP strains causes great concerns about the choice of effective antibiotics to treat nosocomial infections.

In this study, we investigate a fatal outbreak among patient’s hospitalization in ICU in an Iranian hospital during the fifth wave of COVID-19 pandemic with the aim of the molecular tracking for the emerging ST16 CPKP strains responsible for this outbreak.

## Materials and methods

### Outbreak investigation

A retrospective, single-center study including all adult patients with diagnosis of COVID-19 requiring ICU admission and hospitalized at an Iranian hospital in Isfahan was performed. During the fifth wave of COVID-19 pandemic from 14th June to 16th December 2021, our hospital was exclusively allocated to the cure and handling of COVID-19 patients. In late June and early August, we identified several cases of infection due to spread of colistin and carbapenem-resistant *K. pneumoniae* (Col-CRKP) in the ICU. The ICU consists of three wards with 22 beds (a main hall with 18 beds and 4 separate isolated rooms). In addition, there are only two hand washing sinks in the whole hall as well as one hand washing sink in two of the isolated rooms. Before the outbreak described, sporadic cases of infection (one or two cases per year) caused by Col-CRKP isolates were reported among patients. Therefore, it was detected an outbreak that had involved in seven patients. Five patients stayed in the second ward, and one patient hospitalized in each of the first and third wards of the ICU. All patients had an overlapping time in the ICU during this outbreak. All clinical data were extracted from electronic medical records available in the hospital intranet. This project was done based on hospital ethical guidelines as previously approved by Ethical Committee of the Isfahan University of Medical Sciences (approval number IR.ARI.MUI.REC.1402.014).

### Bacterial isolates and antimicrobial susceptibility

Bacterial strains were isolated from the various sample specimens at the clinical microbiology laboratory of the hospital. All isolates were subjected to antibiotic susceptibility testing by Kirby-Bauer disc diffusion method on Mueller Hinton Agar plates (HiMedia, India) against the ceftazidime, cefotaxime, cefepime, amikacin, gentamicin, ciprofloxacin, levofloxcine, piperacillin-tazobactam, ampicillin-sulbactam, imipenem and meropenem as well as nitrofurantoin for urine samples. Minimum inhibitory concentrations (MICs) were determined by E-test (meropenem and imipenem) and broth microdilution (colistin). *E. coli* ATCC 25922 and *Pseudomonas aeruginosa* ATCC 27853 were used as quality control strains. We interpreted these in accordance with the guideline document M100-S30 established by Clinical and Laboratory Standards Institute (CLSI-2017) [10]. Initial screening for detection of carbapenemases was done by the modified carbapenem inactivation method (mCIM) [10].

### Determination of hypermucoviscous phenotype

The hypermucoviscosity phenotype of the isolates was assessed by string test as described previously [11]. Hypermucoviscosity was defined by the formation of viscous strings >5 mm in length when a loop was used to stretch the colony on agar plate [12].

### Capsular serotyping and molecular detection of virulence and resistance genes

Plasmid DNA extraction Mini Kit (FAVORGEN Biotech Corporation, Taiwan) has been used for the detection of genes carried on plasmids. In addition, the boiling method was used for isolation of genomic DNA. Detection of capsular serotype-specific genes including K1, K2, K5, K20, K54, K57 and virulence genes (*iucA*, *peg-344*, *iutA*, *iroB*, *magA*, *kfuB*, *ybtS*, *rmpA* and *alls*) was carried out by PCR assays [2, 13]. The presence of genes encoding beta-lactamases, including ESBLs (*bla*_CTX-M-15_, *bla*_TEM_, *bla*_SHV_) and carbapenemases (*bla*_KPC-1_, *bla*_VIM_, *bla*_IMP_, *bla*_NDM-1_ and *bla*_OXA-48_) genes were investigated by PCR as previously described [14]. Also, detection of genes conferring resistance to colistin was also performed for plasmid genes *mcr-1*, *mcr-2*, *mcr-3*, and *mcr-4* [15].

### Multi-locus sequence typing (MLST)

MLST for all seven isolates was done with seven housekeeping genes (*gapA*, *infB*, *mdh*, *phoE*, *pgi*, *rpoB*, and *tonB*) according to the protocol on the MLST website (https://bigsdb.pasteur.fr/klebsiella/klebsiella.html).

## Results

**Fig. 1:**
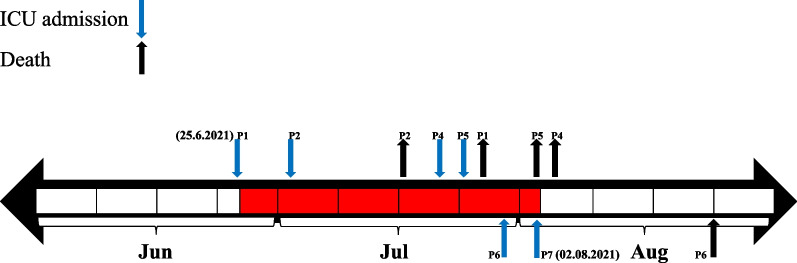
Timeline of the patient’s hospitalization in ICU

**Fig. 2:**
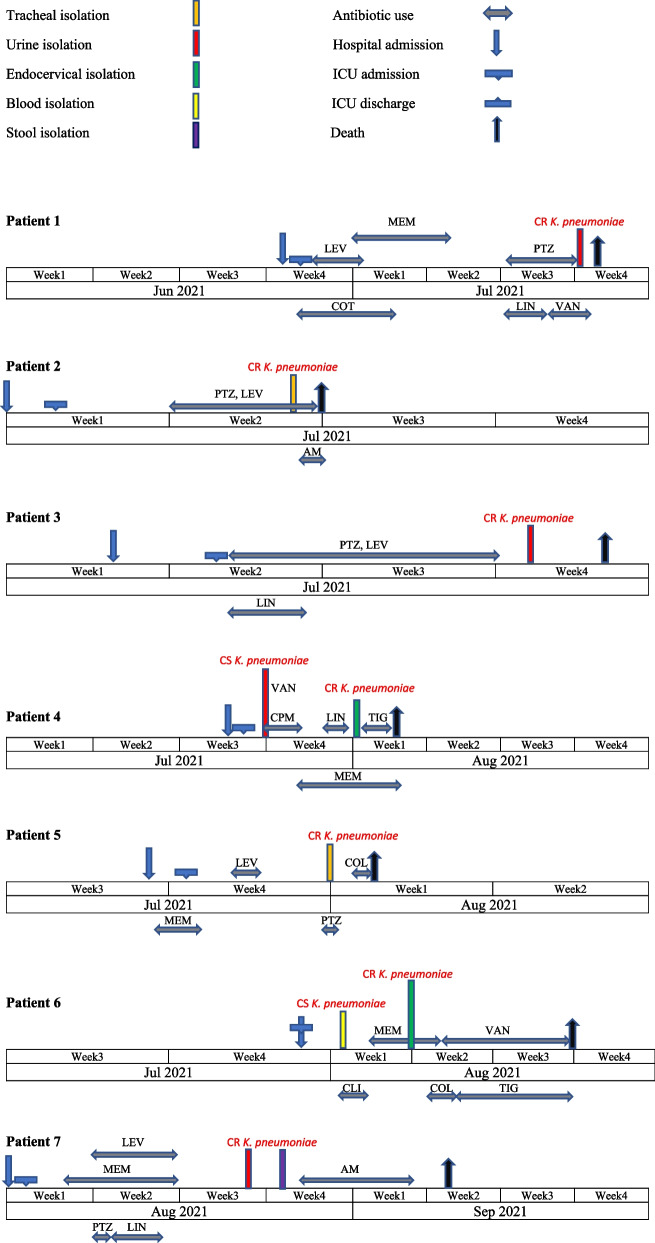
Epidemiology of the carbapenem-resistant *K. pneumoniae* outbreak cases. COT: Co-trimoxazole; MEM: Meropenem; LEV: Levofloxacin; PTZ: Piperacillin*/*tazobactam*;* LIN: Linezolid*;* VAN: Vancomycin; AM: Ampicillin*;* CPM: Cefepime; TIG: Tigecycline; COL: Colistin; CLI: Clindamycin

During the fifth wave of COVID-19 pandemic, the patients were aged 24–75 years with at least 50% pulmonary involvement were admitted to the ICU from 14th June to 16th December 2021. Out of patients, two patients were male and five were female. Of all patients, six cases had underlying diseases such as blood pressure, diabetes, chronic kidney disease, chronic heart disease or pregnancy (Table 1). Timeline of the patient’s hospitalization in ICU are shown in Fig. 1. Following admission to the ICU, they all received antibiotic prophylaxis (Fig. 2), four of the patients also received ACTEMRA® (tocilizumab). The seven patients developed superinfection and all showed various clinical symptoms such as pulmonary edema, purulent discharge, leukocytosis and fever, with at least two symptoms in each patient. The clinical and laboratory profile for the seven Col-CRKP- infected patients are summarized in Table 1. The eight Col-CRKP species isolated from tracheal (*n* = 2), urine (*n* = 3), endocervical (*n* = 2) and stool (*n* = 1) samples of the patients, since all the patients had overlapping stays in the ICU, suggesting that Col-CRKP might be the causative agent of the outbreak. In this study, urinary tract infection, ventilator-associated pneumonia (VAP) and cervicitis infection were reported from three, two and two patients, respectively. Also, colonization with Col-CRKP occurred in patient-7. The mean time from ICU admission to superinfection diagnosis was 13.7 days. All seven patients died of severe infection after Col-CRKP could be recovered from their microbiological samples (Table 1).

**Table 1:** Baseline characteristics, laboratory data, and outcomes of seven COVID-19 patients with Col-CRKP admitted in ICU

Patient	Patient-1	Patient-2	Patient-3	Patient-4	Patient-5	Patient-6	Patient-7
**Age / years**	59	67	40	35	75	24	44
**Gender**	Male	Female	Male	Female	Female	Female	Female
**BMI (kg/m2)**	29.4	31.1	39.2	31.9	31.2	29.4	32
**Symptom duration before hospital admission**	Cough, shortness of breath, chills, diarrhea, anorexia	Shortness of breath	Fever, chills, cough, nausea and vomiting, stuffy nose	Shortness of breath, muscle pain	Cough, shortness of breath, anorexia	Fever, cough, shortness of breath, stuffy nose, conjunctival redness	Fever, shortness of breath, drowsiness, anorexia, muscle pain
**Underlying diseases**	Blood pressure	Blood pressure	–	Pregnant	Diabetes, CHD	Pregnant	CKD
**Steroid**	MTP / DX	MTP	MTP / DXM	MTP	MTP	MTP	MTP / DXM
**Tocilizumab**	Yes	Yes	No	Yes	No	No	Yes
**Remdesivir**	Yes	Yes	No	No	No	No	No
**Invasive procedures**	Urinary catheter, Gastric tube	Urinary catheter, Gastric tube	Urinary catheter	Urinary catheter, Gastric tube, PICC	Urinary catheter, Gastric tube	Urinary catheter, Gastric tube	Urinary catheter, Gastric tube
**Surgery**	No	No	No	Yes	No	Yes	No
**Laboratory data: At ICU admission/ at microbial sampling time**							
White blood cells count (109/ml)	6800 / 1000	9100 / 13,600	12,800 / 14,500	6300 / 12,300	9500 / 12,600	8400 / 19,900	13,000 / 34,900
C-reactive protein (mg/dl)	78 / 86	76 / 3	50 / 4	86 / 5	84 / 54	71 / 54	70 / 2
Neutrophils	93.4 / 90	88.1 / 92.6	85.2 / 85	82.1 / 76.3	87.5 / 95.5	88.9 / 90.5	89.9 / 87.8
Cratinin	0.9 / 0.7	1.1 / 0.8	1.1 / 0.7	0.8 / 0.7	1.3 / 0.9	0.7 / 0.7	0.8 / 0.9
**Fever (≥39) At ICU admission/ at microbial sampling time**	No / Yes	No / No	No / Yes	No / Yes	No / Yes	No / Yes	No / No
**Infection type**	UTI	VAP	UTI	Cervicitis	VAP	Cervicitis	UTI
**Specimen type**	Urine	Tracheal	Urine	Endocervical	Tracheal	Endocervical	Urine and stool
**Mechanical ventilation / days**	No	Yes / 8	No	Yes / 15	Yes / 12	Yes / 18	Yes / 2
**Length of hospital stay, days**	31	14	20	17	14	24	40
**Length of ICU stay, days**	29	12	15	16	12	24	39
**Time from ICU admission to superinfection, days**	26	11	14	13	8	9	15
**Outcomes**	Died	Died	Died	Died	Died	Died	Died

*MTP* Methyl-prednisolone: *DXM* Dexamethasone: *PICC* Peripherally Inserted Central Catheter: *CHD* Chronic heart disease: *CKD* Chronic Kidney disease: *BMI (kg/m2)* BMI, body mass index: *UTI* Urinary tract infection: *VAP* Ventilator-associated pneumonia

### Resistance phenotypes and detection of antimicrobial resistance genes

Results of the antimicrobial susceptibility testing revealed a high-level resistance of *K. pneumoniae* isolates to all tested antibiotics, on the other hand, all clinical isolates were tigecycline susceptible *K. pneumoniae* in line with the EUCAST (Table 2). The MICs of meropenem, imipenem and colistin in seven Col-CRKP isolates were listed in Table 2. The mCIM results showed that all isolates were positive for carbapenemase phenotype.

**Table 2:** Microbiological and genomic characteristics of the Col-CRKP strains

Patients	Isolates	String test	Capsular serotype	Virulence genes	MLST	Resistance determinant genes	mCIM	Resistance profile	MIC of antimicrobials (μg/ml)		
									MEM	IMI	COL
Patient-1	P-1	Neg	ND	ND	ST16	*bla*_CTX-M-15_, *bla*_SHV_, *bla*_TEM_, *bla*_NDM-1_, *bla*_OXA-48_	Pos	CAZ, CPM, PTZ, ASM, AM, GM, CIP, LEV, NIT	≥32	≥32	12
Patient-2	P-2	Pos	K20	*iucA*, *ybtS*	ST16	*bla*_CTX-M_-_15_, *bla*_SHV_, *bla*_TEM_, *bla*_NDM-1_, *bla*_OXA-48_	Pos	CAZ, CPM, PTZ, ASM, AM, GM, CIP, LEV	≥32	≥32	8
Patient-3	P-3	Neg	ND	ND	ST11	*bla*_OXA-48_	Pos	CAZ, CPM, PTZ, ASM, AM, GM, CIP, LEV, NIT	≥32	16	12
Patient-4	P-4	Neg	ND	ND	ST16	*bla*_CTX-M_-_15_, *bla*_SHV_, *bla*_TEM_, *bla*_NDM-1_, *bla*_OXA-48_	Pos	CAZ, CPM, PTZ, ASM, AM, GM, CIP, LEV	≥32	≥32	12
Patient-5	P-5	Neg	ND	ND	ST16	*bla*_CTX-M-15_, *bla*_SHV_, *bla*_TEM_, *bla*_NDM-1_, *bla*_OXA-48_	Pos	CAZ, CPM, PTZ, ASM, AM, GM, CIP, LEV	≥32	≥32	16
Patient-6	P-6	Neg	ND	ND	ST16	*bla*_CTX-M-15_, *bla*_SHV_, *bla*_TEM_, *bla*_NDM-1_, *bla*_OXA-48_	Pos	CAZ, CPM, PTZ, ASM, AM, GM, CIP, LEV	≥32	≥32	12
Patient-7	P-7	Neg	ND	ND	ST16	*bla*_SHV_, *bla*_NDM-1_	Pos	CAZ, CPM, PTZ, ASM, AM, GM, CIP, LEV, NIT	≥32	≥32	12
	P-8	Neg	ND	ND	ST16	*bla*_SHV_, *bla*_NDM-1_	Pos	CAZ, CPM, PTZ, ASM, AM, GM, CIP, LEV, NIT	≥32	≥32	12

*Neg* Negative, *Pos* Positive, *ND* Not detected, *iucA* Aerobactin, *ybt* Yersiniabactin, *MLST* Multilocus sequence typing, *mCIM* Modified carbapenem inactivation method, *Pos* Positive, *CAZ* Ceftazidime, *CPM* Cefepime, *PTZ* Piperacillin/tazobactam, *ASM* Ampicillin/sulbactam, *AM* Amikacin, *GM* Gentamicin, *CIP* Ciprofloxacin, *LEV* Levofloxacin, *NIT* Nitrofurantoin, *MEM* Meropenem, *IMI* Imipenem, *COL* Colistin, *MIC* Minimal inhibitory concentration

Among eight Col-CRKP isolates only one isolate was positive for the string test and identified as hypervirulent *K. pneumoniae* (Table 2). MLST analysis revealed that the eight Col-CRKP isolates belonged to two STs. Seven isolates were identified as ST16 that five isolates co-carried *bla*_OXA-48_ and *bla*_NDM-1_ and two isolate carried only *bla*_NDM-1_ gene. Also, one isolate that recovered from patient-2 belonged to ST11, which only carried the *bla*_OXA-48_ gene. All Col-CRKP strains carried at least one ESBL genes except one isolate that belonged to ST11. The *bla*_SHV_ gene was the most prevalent ESBL gene (7/8), followed by *bla*_CTX-M-15_ and *bla*_TEM_ (5/8) (Table 2). All isolates were negative for the *mcr-1*, *mcr-2*, *mcr-3*, and *mcr-4* genes.

### Capsular genotyping and detection of virulence genes

Capsular genotyping (K genotyping) of isolates showed that capsular serotype K20 was detected in only two isolates. PCR for virulence-associated genes revealed that *iucA* and *ybtS* were identified in only two and one isolate, respectively. The other virulence factor genes were not detected in any of the strains.

## Discussion

Our results show the emergence of ST16 Col-CRKP strains that caused fatal hospital infections. In the present study, all seven patients were infected between late June and early August. Outbreak of Col-CRKP, it probably indicates a near-patient environmental source, pointing to poor hand hygiene and lack of compliance with device-related bundle care protocols as contributing factors. It is possible that Col-CRKP isolate was transferred to patients four and six through the gynecologist during vaginal ultrasound or through the nurse’s assistant during vaginal care or stool cleaning. Similar to previous reports [16, 17], since the implementation of infection prevention and control (IPC) procedures in this ICU, no fatal infections due to ST16 CRKP have occurred until the end of the fifth wave of COVID-19 pandemic. However, more evidence is needed to confirm that this IPC policy is effective in preventing CRKP infections in our ICU.

High mortality rate (approximately 69%) in bloodstream infections due to Col-CRKP was also reported in a study in India [18].

According to our results, six of seven COVID-19 patients had normal WBC counts at ICU admission, while six patients had leukocytosis and one patient had leukopenia at microbial sampling time. In addition, none of the patients had fever at the time of hospitalization in the ICU, but five of the patients had fever at microbial sampling time, which could be resulted from bacterial co-infection in patients. These results are consistent with He′s study in China [19]. In the present study, the median days from ICU admission to bacterial growth was 13.7 days [8–26]. It is well known that most hospitalized severe COVID-19 patients are prescribed steroids, undergo invasive procedures and sometimes have a prolonged ICU stay, rendering them vulnerable to be at higher risk of secondary infections [20]. Five patients received tocilizumab and seven patients received at least one steroid, also, invasive tools were used for all patients.

Similar to other studies [21], we found that patients were often treated with early empiric antibacterial. Piperacillin-tazobactam and meropenem were the most commonly prescribed antibiotics. Antimicrobial susceptibility testing in our study confirmed resistance to all antibiotics in all eight isolates. Since none of the available antibiotics was effective in treating infections caused by ST16 Col-CRKP strains, we have implemented a new IPC policy to control the outbreak in the hospital. And it also shows the urgent need for novel β-lactam agents (i.e., ceftazidime-avibactam and cefiderocol) to treat patients with this infection. Notably, all Col-CRKP strains carried at least one carbapenemase gene (*bla*_NDM-1_ or *bla*_OXA-48_). We also found that five out of eight isolates co-carried *bla*_NDM-1_ and *bla*_OXA-48_, a finding consistent with that reported previously in Iran [14, 22, 23]. This highlights that isolates with *bla*_NDM-1_ and *bla*_OXA-48_ genes continue to be a problem in Iran. Col-CRKP with pandrug-resistant and XDR phenotype co-producing *bla*_NDM-1_ and *bla*_OXA-48_ carbapenamases have been reported to cause severe nosocomial infections in several countries [18, 24, 25]. The previously reported cases of *bla*_NDM-1_ and *bla*_OXA-48_–harboring *K. pneumoniae* in Iran were mainly serotypes ST11, ST147 and ST893 [4, 22, 26], while ST16 was the dominant epidemic serotypes in our study. Since ST11 is the major clone of CRKP in Iran, the prevalence of ST16 is very significant, especially as it was related to hospital-acquired infections.

Thus, ST16 may be a high-risk, CPKP clone actively disseminating across our hospital, with related outbreaks being reported in Thailand [27].

Outbreaks of *K. pneumoniae* ST16 carrying carbapenemase and ESBL genes, have recently been sporadically reported. In the last years, numerous hospital surveillance programs from different countries reported ST16 and ST11 carrying different antimicrobial resistance profiles. *K. pneumoniae* ST16 associated with NDM-1, CTX-M-15, and OXA-232 caused infections in a hospital in Italy [28], and in Thailand a carbapenem-resistant ST16 clone co-producing NDM-1 and OXA-232 was also reported. Hypervirulence genes (*iucA*, *ybtS*) were identified in only one ST16 isolate in our study, contrary to our findings, Abe et al. in Thailand reported that all the ST16 isolates are non-hypervirulent [29]. In our previous study, a case of infection caused by *K. pneumoniae* ST16 was reported, unlike this study, the patient was treated with tigecycline [30].

## Conclusion

In conclusion, we reported the fatal outbreak of an XDR *K. pneumoniae* ST16 in a hospital in Iran, which carried the carbapenemase genes in combination with ESBLs. To the best of our knowledge, this study is the first to report on the NDM-1 and OXA-48-producing hypervirulent *K. pneumoniae* ST16-K20 causing fatal VAP. Therefore, it is enormously important to strengthen antibiotic control to prevent the development of antimicrobial resistance and to emphasize infection control measures are needed to prevent Col-CRKP from further disseminating in hospital settings and the community.

## Data Availability

The datasets used and analyzed during the current study are available from the corresponding author upon reasonable request.

## Abbreviations

Col-CRKP: Colistin and carbapenem-resistant *Klebsiella pneumoniae*
MLST: Multi-locus sequence typing
MCR: Mobilized colistin-resistance
ICU: Intensive care unit
CRKP: Carbapenem-resistant *K. pneumoniae*
CPKP: Carbapenemase-producing *K. pneumoniae*
XDR: Extensively drug-resistant
MIC: Minimum inhibitory concentrations
CLSI: Clinical and Laboratory Sltandards Institute
eCIM: Modified carbapenem inactivation method
VAP: Ventilator-associated pneumonia
IPC: Infection prevention and control.
